# The UCSC Genome Browser database: 2015 update

**DOI:** 10.1093/nar/gku1177

**Published:** 2014-11-26

**Authors:** Kate R. Rosenbloom, Joel Armstrong, Galt P. Barber, Jonathan Casper, Hiram Clawson, Mark Diekhans, Timothy R. Dreszer, Pauline A. Fujita, Luvina Guruvadoo, Maximilian Haeussler, Rachel A. Harte, Steve Heitner, Glenn Hickey, Angie S. Hinrichs, Robert Hubley, Donna Karolchik, Katrina Learned, Brian T. Lee, Chin H. Li, Karen H. Miga, Ngan Nguyen, Benedict Paten, Brian J. Raney, Arian F. A. Smit, Matthew L. Speir, Ann S. Zweig, David Haussler, Robert M. Kuhn, W. James Kent

**Affiliations:** 1 Center for Biomolecular Science and Engineering, CBSE, UC Santa Cruz, 1156 High Street, Santa Cruz, CA 95064, USA; 2 Institute for Systems Biology, Seattle, WA 98109, USA; 3 Howard Hughes Medical Institute, UCSC, Santa Cruz, CA 95064, USA

## Abstract

Launched in 2001 to showcase the draft human genome assembly, the UCSC Genome Browser database (http://genome.ucsc.edu) and associated tools continue to grow, providing a comprehensive resource of genome assemblies and annotations to scientists and students worldwide. Highlights of the past year include the release of a browser for the first new human genome reference assembly in 4 years in December 2013 (GRCh38, UCSC hg38), a watershed comparative genomics annotation (100-species multiple alignment and conservation) and a novel distribution mechanism for the browser (GBiB: Genome Browser in a Box). We created browsers for new species (Chinese hamster, elephant shark, minke whale), ‘mined the web’ for DNA sequences and expanded the browser display with stacked color graphs and region highlighting. As our user community increasingly adopts the UCSC track hub and assembly hub representations for sharing large-scale genomic annotation data sets and genome sequencing projects, our menu of public data hubs has tripled.

## INTRODUCTION

The UCSC Genome Browser database (1,2) is a large collection of genome assemblies and annotations for vertebrate and selected model organisms that has been under active development since 2000. At present, the database contains 160 genome assemblies representing 91 species.

Accompanying the genomes are details of the sequencing and assembly, gene models from RefSeq (3,4), GENCODE (5), Ensembl (6,7) and UCSC (8), transcription evidence from GenBank (9) and other sources, epigenetic and gene regulatory annotation including comprehensive data sets from the ENCODE project (10), comparative genomics and evolutionary conservation annotation, repetitive element identification from RepeatMasker and other sources, biomedical annotations encompassing phenotype, literature and genome variants from dbSNP (11), the 1000 Genomes project (12) and other sources and comprehensive annotation of repetitive elements in the genome. Experimental data are available together with analysis and summary results and modeling efforts. Publications are linked to genomic regions via sequences and gene variants (13). UCSC provides a portal for data from the Neanderthal and Denisova early hominid genome projects (14,15). We host all data produced by the ENCODE Consortium during the first 11 years of the project when UCSC served as the Data Coordination Center (16).

While the core function of the database is to support visualization via the Genome Browser, the UCSC bioinformatics group provides a diverse set of additional web-based and command-line tools to facilitate use of these data by a broad user community, ranging from college-level biology students to specialists in genetic, genomic and biomedical research. Notable bioinformatics tools developed at UCSC include the BLAT sequence alignment tool (17) and the liftOver/chain/net inter-assembly genome coordinate converter (18).

Here we share a brief overview of key features of the Genome Browser, cover in-depth new features of the browser database and software and indicate some future directions. We conclude with an addendum describing the process we use to create a new genome browser. Readers who are already familiar with the Genome Browser software and data may want to skip to the ‘New Data in the Genome Browser’ section.

## KEY FEATURES OF THE BROWSER

### Display and navigation

The UCSC Genome Browser provides a full-featured and highly flexible display for genomic annotations via a high-performance dynamic web interface. All annotation data sets in the browser are formatted and configured for visual display at varying levels of resolution. The user interface allows zooming to genomic regions of arbitrary size, from whole chromosome to base-level view. A range of visibility modes, from detailed views to compact density graphs, provide customized viewing of multiple annotations in context. Annotation track menus, a configuration page, and a search tool are available to locate data of interest. Navigation to genomic regions of interest can be accomplished using a variety of location identifiers, including genome coordinates, gene names, chromosome band identifiers and dbSNP IDs. The main browser display can be configured with mouse actions that zoom in, pan across or highlight regions. Visibility and ordering of annotation tracks on the screen are configurable via mouse click and drag actions. Details about genes, SNPs and other annotation items are displayed on track detail pages. Track description pages explain display conventions, the methods used to create the annotation and applicable credits and references. A configured browser view, including any user data tracks, can be saved as a named ‘session’ for later reference and to share with collaborators.

### User data in the browser

User data can be viewed together with annotations hosted at UCSC via a variety of mechanisms. A small annotation file can be uploaded and viewed in the browser as a ‘custom track’. Larger files can be formatted to support remote access, using tools available at UCSC, so they can be viewed using the custom track mechanism. Collections of data sets can be organized with configuration and documentation as ‘track data hubs’, which can be viewed locally or shared (19). A newer related feature, ‘assembly hubs’, allows use of UCSC Genome Browser technology to visualize a user-hosted genome assembly and annotations via remote access. Assembly hub developer instructions are available alongside those for track hubs at http://genome.ucsc.edu/goldenPath/help/hgTrackHubHelp.html. UCSC makes track and assembly hubs of general interest available to users by adding them to the site's ‘public hubs’ menu (http://genome.ucsc.edu/cgi-bin/hgHubConnect). If full data privacy or development of new visualizations is desired, a browser mirror or a Genome Browser in a Box (GBiB) instance can be installed (free for academic and other non-profit use). User data loaded via these mechanisms are accessible to the UCSC Table Browser and other tools described below.

### Other tools

The UCSC Genome Browser website provides additional tools to access the database and complement the Genome Browser. The web-based Table Browser enables data mining of the Genome Browser database. Users can retrieve data from database tables, filter and intersect with other tables and obtain output in a variety of formats, including as a custom track in the browser. Output also can be directed to outside data analysis platforms such as Galaxy (20), GenomeSpace and GREAT (21).

The Variant Annotation Integrator (VAI) allows users to upload variants and see potential functional effects on user-selected transcript sets and regulatory data sets in the Genome Browser database (e.g. ENCODE, COSMIC or conserved elements). Users can upload variants in a variety of formats: dbSNP rs identifiers, VCF and pgSNP format and obtain output as HTML views or tab-separated text in Ensembl Variant Effect Predictor format (22).

Other web-based tools include the Gene Sorter, Genome Graphs and Visigene. The popular ‘liftOver’ tool, which translates genomic coordinates of a data file between assemblies using the UCSC chain/net annotation, is available in both web and command-line forms. We also provide the widely used BLAT sequence alignment tool in both forms.

Tools for formatting data sets for hubs and other popular command-line tools from UCSC are available as prebuilt binaries for Linux and Mac OSX (http://hgdownload.soe.ucsc.edu/downloads.html#utilities_downloads). The source code for all programs used at UCSC to build genome browsers is freely available for non-commercial use from our downloads page as a zip file or via git (http://genome.ucsc.edu/admin/git.html). We have set up a secure online web store at https://genome-store.ucsc.edu/ where GBiB, the Genome Browser binaries and source code, and the liftOver tool can be downloaded or purchased. For the convenience of our users we support mirror sites, including a European mirror (http://genome-euro.ucsc.edu) hosted by Bielefeld University in Germany.

## NEW DATA IN THE GENOME BROWSER

### New genome assemblies

#### Human genome

The highlight of the year for the Genome Browser project was the release of a UCSC browser for the first new human genome assembly in 4 years. The December 2013 human genome assembly (GenBank GCA_000001405.15) is produced by the Genome Reference Consortium (NCBI, EMBL-EBI, Sanger Institute, and Washington University) and versioned GRCh38 (23,24). To reduce user confusion going forward, UCSC has adopted the GRC version number, i.e. the UCSC identifier for this assembly is hg38, skipping ahead from the hg19 version number of the previous human assembly.

The new assembly includes many changes from the GRC, including several thousand corrected bases in both coding and non-coding regions, centromere sequence representation, updated mitochondrial reference sequence from MITOMAP and an extensive data set for sequence variation presented as alternate reference sequences. Several well-known variant regions in the human genome, such as the immunoglobulin heavy chain locus (IGH), have been retiled to ensure the reference assembly provides haplotypic representation. Additionally, incorrectly assembled regions in the previous build (GRCh37/hg19) within 1q21, 10q11 and chromosome nine pericentromeric regions have been greatly improved and retiled through the new release. Overall, more than 100 assembly gaps have been closed or reduced with available data from other genome sequencing projects. These collective changes observed between the GRCh37/hg19 and the new reference assemblies are presented on the Genome Browser as the ‘Hg19 Diff’ mapping and sequencing track. The track annotation allows users to clearly differentiate new contigs from those repurposed to improve the assembly or modified to correct sequence errors. A more extensive listing of assembly updates can be referenced directly through the GRC website (http://www.ncbi.nlm.nih.gov/projects/genome/assembly/grc/human/).

In addition to sequence updates in the primary chromosome assemblies, GRCh38 includes a large number of alternative reference sequences. These 261 ‘alternate loci scaffolds’ represent 178 human chromosomal regions that exhibit sufficient variability to prevent adequate representation by a single sequence (http://www.ncbi.nlm.nih.gov/projects/genome/assembly/grc/human/). For example, the human leukocyte receptor complex (LRC/KIR, located on 19q13.4), which comprises genes encoding natural killer receptors known to be highly variant in the human population, has the largest number of alternate sequence representations. The annotated alternate sequence assemblies can be accessed from the ‘Sequences’ link on the GRCh38 gateway page (http://genome.ucsc.edu/cgi-bin/hgTracks?db=hg38&chromInfoPage=), enabling user alignments and custom track applications similar to the main chromosome assemblies.

One of the biggest innovations in the GRCh38 assembly is the replacement of megabase-sized gaps in human centromere regions with satellite sequence reference models. These models are generated using second-order Markov models of local ordering and frequency of repeat variants through an analysis of Sanger reads from the HuRef sequencing project (25). In the absence of these sequences, roughly 3% of the human genome represented by alpha satellite DNA is often misassigned to sites in the reference assembly, inflating enrichment peak signals and accounting for current blacklisted regions (or regions typically masked for sequence-based analysis).

To accommodate next-generation sequencing read alignment pipelines, the GRCh38 assembly offers an analysis set in which several regions have been masked to improve read mapping. To avoid false mapping of reads, duplicate copies of centromeric arrays on several chromosomes have been hard-masked (represented as a string of ‘N’ characters). The two pseudoautosomal regions on chromosome Y have also been hard-masked, and the Epstein–Barr virus sequence has been added as a decoy to attract contamination in samples. Two versions of the analysis set are available on the UCSC Genome Browser downloads page: one without the alternate chromosomes from this assembly and one that includes them.

To expedite the availability of this new assembly, UCSC released the hg38 browser in March 2014 with a basic set of annotations, retaining the hg19 browser as the default human genome on our Gateway page. The annotations include extensive mapping and sequencing tracks, gene models, prediction and curation from multiple sources (including UCSC, GENCODE, CCDS and Genscan), GenBank mRNA and EST alignments and the biomedically oriented GeneReviews and OMIM Genes literature tracks. Throughout the year we have continued to expand the annotation set. Larger annotations from external sources (e.g. dbSNP) and those requiring lengthy computation (large multi-species sequence alignment and conservation) will be released as they become available. Additionally, users will be able to identify reference sequence updates, or sequence patches, as they become available on a UCSC hosted ‘patch track’, similar to that implemented in hg19. The initial hg38 browser comparative genomics annotation shows a genome alignment of seven mammalian species with conservation scores.

#### Rat genome

The rat genome also received an updated browser this year. In July 2014 the Rat Genome Sequencing released RGSC 6.0, the first new assembly in 2 years. This assembly includes sequence from and partial assembly of chromosome Y. UCSC released a genome browser with basic annotation set shortly thereafter. The previous assembly (March 2012, rn5), which remains the default rat genome in the browser, was augmented this year to include a 13-species comparative genomics track with conservation scores produced by two methods (phastCons and phyloP).

#### Ebola virus genome

The 2014 Ebola epidemic in West Africa has stirred international response and renewed efforts to develop effective preventative and treatment options. In response to a request for help from vaccine researchers, in September 2014 we fast-tracked an Ebola genome browser (26) showing all available viral sequences from previous outbreaks and the recently published 99 genome sequences from the 2014 outbreak (27). Informational materials are available from a portal at http://genome.ucsc.edu/ebolaPortal/index.html.

#### Other species

UCSC has created genome browsers for three newly sequenced animals this year. Chinese hamster (*Cricetulus griseus*), a native to deserts of Mongolia and China, is of biomedical significance as the source of the CHO (Chinese hamster ovary) cell lines commonly used as production hosts for therapeutic proteins. The elephant shark (*Callorhinchus milii*), a cartilaginous fish native to temperate waters off Australia and New Zealand, is the largest non-mammalian vertebrate. Analysis of the 9-GB draft assembly reported this shark to be the slowest evolving of known vertebrates, displaying extensive conservation of synteny with tetrapods and an unusual adaptive immune system (28). The minke whale (*Balaenoptera acutorostrata*) is the most abundant baleen whale. Analyses of the 2.4-GB genome reported clues to mammalian adaptation to the ocean environment, especially hypoxia during deep dives (29). Other species with new browsers under development for public release are bald eagle (*Haliaeetus leucocephalus*), Chinese pangolin (*Manis pentadactyla*) and hooded crow (*Corvus cornix cornix*).

The browsers for eight species were updated with new assemblies this year, including five from the Broad Institute's 29 mammal comparative genomics project (30) (alpaca, hedgehog, pika, shrew and tenrec) and an updated cow assembly (bosTau8, UMD 3.1.1) from the University of Maryland that removed 138 contigs representing contaminant DNA from the UMD 3.1 (bosTau6) assembly. The complete list of genome assemblies built at UCSC this year for public release can be found in Table 1.

**Table 1.** New genome browsers

Common Name	Scientific name	Sequencing Center	Center Date and Assembly ID	UCSC ID
***New species***				
Chinese hamster	*Cricetulus griseus*	Beijing Genomics Institute-Shenzen	July 2013 C_griseus_v1.0	criGri1
Elephant shark	*Callorhinchus milii*	Institute of Molecular & Cell Biology, Singapore	December 2013 Callorhinchus_milii-6.1.3	calMil1
Minke whale	*Balaenoptera acutorostrata scammoni*	Korea Ocean Research & Development Institute	October 2013 BalAcu1.0	balAcu1
Ebola virus	*Filoviridae ebolavirus*	Broad Institute	Jun 2014 West Africa 01 EBOV/G3683/KM034562.1	eboVir3
^a^Bald eagle	*Haliaeetus leucocephalus*	The Bald Eagle Consortium	August 2014 Haliaeetus_leucocephalus-4.0	halLeu1
^a^Chinese pangolin	*Manis pentadactyla*	Washington University	August 2014 m_pentadactyla-1.1.1	manPen1
^a^Hooded crow	*Corvus cornix cornix*	Uppsala University	August 2014 Hooded_crow_genome	corCor1
Ebola virus	*Filoviridae ebolavirus*	Broad Institute	Jun 2014 West Africa 01 EBOV/G3683/KM034562.1	eboVir3
***Updated species***				
Alpaca	*Vicugna pacos*	Broad Institute	March 2013 Vicugna_pacos-2.0.1	vicPac2
Hedgehog	*Erinaceus europaeus*	Broad Institute	May 2012 EriEur2.0	eriEur2
Human	*Homo sapiens*	Genome Reference Consortium	December 2013 GRCh38	hg38
Pika	*Ochotona princeps*	Broad Institute	May 2012 OchPri3.0	ochPri3
Rat	*Rattus norvegicus*	Rat Genome Sequencing Consortium	July 2014 Rnor_6.0	rn6
Sheep	*Ovis aries*	International Sheep Genome Consortium	August 2012 Oar_v3.1	oviAri3
Shrew	*Sorex araneus*	Broad Institute	August 2008 sorAra2	sorAra2
Tenrec	*Echinops telfairi*	Broad Institute	November 2012 echTel2	echTel2
Zebra Finch	*Taeniopygia guttata*	Washington University	February 2013 taeGut2	taeGut2
^b^Cow	*Bos taurus*	University of Maryland	June 2014 Bos_taurus_UMD_3.1.1	bosTau8

^a^In progress.

^b^Expected release by January 1, 2015.

Additional genome browsers for species not integrated into the core of our site were made available to our users as public assembly hubs, including three plants: *Arabidopsis thaliana* (a model plant), *Brassica rapa* (varied subspecies in the human diet) and *Ricinus communis* (castor oil plant).

### New and updated genome annotation

This year UCSC released 233 new or updated browser tracks on existing assemblies and 13 public track and assembly hubs. Public hubs have become an important way for our user community to share annotations, and we provide development guidance and quality review for public hubs to maximize usability and performance. We have developed guidelines to foster consistency in data presentation and we require appropriate documentation to accompany the data. More details regarding new data sets hosted natively at UCSC (listed in Table 2) or made available via public hub (Tables 3 and 4) are presented below.

**Table 2.** New annotation tracks in the Genome Browser

Annotation track name	Track group	Assembly
Human genome		
Centromere locations	Mapping	hg38
Chromosome Bands Localized by FISH Mapping Clones	Mapping	hg38
GRC Incident Database	Mapping	hg38
Locus Reference Genome (LRG) Regions	Mapping	hg19, hg38
Gene Annotations from GENCODE Version 19 (Ensembl 76)	Genes	hg19, hg38
Ensembl Genes 74, 75	Genes	hg19
UCSC Genes	Genes	hg38
ClinVar Variants	Phenotype	hg19
DNA Sequences in Web Pages Indexed by Bing.com/Microsoft Research	Phenotype	hg19
Genetic Association Studies of Complex Diseases and Disorders (update)	Phenotype	hg19
GeneReviews	Phenotype	hg38
OMIM Genes	Phenotype	hg38
Genome Segmentations from ENCODE	Regulation	hg19
Transcription Factor ChIP-seq (161 factors) from ENCODE with Factorbook Motifs	Regulation	hg19
UCSF Brain DNA Methylation	Regulation	hg19
Database of Genomic Variants: Structural Variation (update)	Variation	hg18, hg19
NHLBI GO Exome Sequencing Project (ESP)—Variants from 6503 Exomes	Variation	hg19
Simple Nucleotide Polymorphisms (dbSNP 141)	Variation	hg19
SNP 135/137 (corrections)	Variation	hg19
Alpaca, American alligator, Cat, Chinese hamster, Hedgehog, Pika, Sheep, Shrew, Tenrec, Zebra finch Chain/Net tracks	Comparative	hg19
Multiz Alignment & Conservation (seven species)	Comparative	hg38
Vertebrate Multiz Alignment & Conservation (100 Species)	Comparative	hg19
Multiz Alignment & Conservation (seven species)	Comparative	hg38
phastBias gBGC predictions	Comparative	hg18, hg19
Mouse genome		
INSDC Chromosome Names	Mapping	mm10
MGI Quantitative Trait Loci	Mapping	mm10
Ensembl Genes v74, v75	Genes	mm10
Ensembl Genes v73	Genes	mm9
GENCODE Genes vM2	Genes	mm10
UCSC Genes	Genes	mm10
DNA Sequences in Web Pages Indexed by Bing.com/Microsoft Research	Literature	mm9
FaceBase 24 Sample Types	Expression	mm10
SNP 138	Variation	mm10
Alpaca, Human, Shrew Chain/Net tracks	Comparative	mm10
Vertebrate Multiz Alignment and Conservation (60 species)	Comparative	mm10
Other genomes		
Accession at International Nucleotide Sequence Database Collaboration (INSDC)	Mapping	46 species
Ensembl Genes v74	Genes	17 species
Ensembl Genes v75	Genes	41 species

**Table 3.** Public track hubs in the Genome Browser

Hub	Source	Assemblies
In progress		
Ensembl Regulatory Build	Ensembl	hg19, hg38
FaceBase Hub	FaceBase Consortium	mm9, mm10, hg18, hg19
FANTOM5 CAGE RECLU Analysis	RIKEN Institute	hg18, hg19
Released September 2013–August 2014		
FANTOM5 CAGE	RIKEN Institute	hg19
Zebrafish Genomics	Shawn Burgess Lab, NHGRI	danRer7
Broad Improved Canine Annotation v1	Vertebrate Biology Group, Broad Inst	canFam3
Ultraconserved Elements	David Haussler Lab, UCSC	hg19
Roadmap Epigenomics Integrative Analysis Hub	Roadmap Epigenomics Project, WUSTL	hg19
McGill Epigenomics	McGill Epigenomics Mapping Centre	hg19
454K562 and HelaS3 RNA-seq	Mike Snyder Lab, Stanford	hg19
BCGSC Epigenomics	Centre for Epigenomic Mapping Technologies, Vancouver	hg19
Older		
Blueprint Epigenomics	European Bioinformatics Institute	hg19
Cancer Genome polyA Site & Usage	University of Pittsburgh and Helicos Biosciences	hg19
Brain H3K4me3 ChIP-seq	Weng Lab, UMass Medical School	hg19
Roadmap Epigenomics Data Complete Collection at Wash U VizHub	Roadmap Epigenomics Project, WUSTL	hg19
miRcode microRNA Sites	Larsson Lab, Sahlgrenska Academy, University of Gothenburg, Sweden	hg19
Translation Initiation Sites	Hampe Lab, University Hospital Schleswig-Holstein, Kiel, Germany	hg19
SDSU Exon Array Gene Expression	Bioinformatics group at South Dakota State University	hg19, mm9, rn4
DNA Methylation	Andrew Smith Lab, USC	hg18, hg19, mm9, rheMac3

**Table 4.** Public assembly hubs in the Genome Browser

Hub name	Source	Assemblies
In progress		
Croc and Bird Hub	David Haussler Lab, UCSC	24 bird, turtle, and crocodilian genomes, plus 22 reconstructed ancestral genomes
Released this year		
EcoliCompHub	David Haussler Lab, UCSC	57 *E. coli* strains, plus nine other bacterial species
EcoliCompHubWtDups	David Haussler Lab, UCSC	57 *E. coli* strains, plus nine other bacterial species
Plants	UCSC Genome Bioinformatics Group	araTha1 (*A. thaliana*), braRap1 (*B. rapa*), ricCom1 (*R. communis*)
ThorntonHub	Kevin Thornton lab, UC Irvine	*D. simulans*
Released last year		
DNA Methylation	Andrew Smith Lab, USC	tair10 (*A. thaliana*)

#### Gene sets

##### UCSC genes

The UCSC Genes set includes both protein-coding and non-coding RNA genes from RefSeq, GenBank, the tRNA Genes track (31) and RFAM (32). The gene set is built for the human and mouse assemblies at UCSC using a multi-step pipeline that utilizes BLAT alignments of RefSeq and GenBank RNAs, gene models from the Consensus CDS project, along with evidence from comparative genomics (http://genome.ucsc.edu/cgi-bin/hgTrackUi?db=hg19&g=knownGene). We typically update the UCSC genes on the latest assemblies every 9–12 months. This year we released a new UCSC Genes set for the GRCh38/hg38 human assembly and updated the gene set for the GRCm38/mm10 mouse assembly. The human UCSC Genes increased by 21 218 transcripts to a total of 104 178 transcripts. The number of genes increased from 31 848 to 48 424. In the mouse UCSC Genes set, the number of transcripts increased from 59 121 to 61 642, with 97% remaining the same between versions.

##### GENCODE genes

The GENCODE project provides high-quality manual curation of evidence-based automated gene predictions for human and mouse genomes. UCSC hosts GENCODE gene sets with a customized display and configuration options. This year we updated the human (hg19) GENCODE track to the December 2013 release, V19 (corresponding to Ensembl 74). As GENCODE for the new human genome assembly was not yet available, we created an interim GENCODE track for hg38 this year by ‘lifting’ the hg19 coordinates for GENCODE V19 to hg38. The first track of GENCODE Genes for mouse was loaded at UCSC this year—GENCODE VM2 (July 2013, Ensembl 74) for mm10. By the end of 2014 we expect to update the hg38 gene set with GENCODE V20 (June 2014) native annotations, to replace the lifted V19 set and update the mouse gene set to GENCODE VM3 (June 2014).

##### Ensembl genes

The Ensembl gene sets on the browsers for 41 species, including human and mouse, were updated to Ensembl 75 (February 2014). GENCODE and Ensembl gene sets for the human genome have converged; however, for user convenience, UCSC maintains a separate Ensembl track on the human hg19 browser in the same format as Ensembl genes in other organisms.

#### Phenotype, literature and variation

Several large collections of human phenotype and variation data were incorporated into the Genome Browser this year. The ‘ClinVar Variants’ track (hg19) displays variants in the NCBI ClinVar database. ClinVar is a free, public archive reporting relationships among human variations and phenotypes, with supporting evidence (33). Variants from 6503 exomes produced by the NHLBI GO Exome Sequencing Project, analyzed and released by the Exome Variant Server (http://evs.gs.washington.edu/EVS) (34), are displayed in the new ‘EVS Variants’ track on hg19. A public hub from the FaceBase Consortium displays tracks of copy number variation in trios with cleft palate offspring (hg18, hg19) as well as related enhancer assay results in mice (mm9, mm10).

Data mining of the literature formed the basis for the new ‘Web Sequences’ track. UCSC and Microsoft Research collaborated to ‘BLAT the web’ by implementing a DNA sequence detector and processing 30 days of Bing web crawler updates covering roughly 40 billion web pages. The resulting sequences were then mapped to the appropriate genome. Human (hg19), mouse (mm9), rat (rn4) and 10 other species browsers host this annotation.

This year the SNP138 build of NCBI dbSNP was loaded as tracks in the current mouse, cow, pig and chicken genome browsers. We expect to have the SNP141 data available for the human hg19 and hg38 browsers by the end of 2014.

#### Gene expression and regulatory elements

##### Transcriptome

UCSC has added two public track hubs of human (hg19) and mouse (mm9) promoter maps produced by the FANTOM5 project using CAGE and single-molecule sequencing technologies (35). The project assayed 988 human samples and 394 mouse samples representing hundreds of cell types and tissues.

##### Epigenome and transcriptional regulation

Five new tracks of ENCODE summary and integrative analysis results were released by UCSC this year. These include DNase Hypersensitivity clusters and ‘master sites’ based on assays covering 125 cell types, Transcription Factor Binding Sites covering 161 factors and Genome Segmentations for six broadly studied cell types. Motif localizations from the Factorbook ENCODE annotation (36) are now displayed together with the ENCODE TF binding peak clusters.

A wealth of additional epigenomics data including DNA methylation and histone maps can be viewed at UCSC from three new public track hubs contributed by outside groups: Ensembl Regulation, McGill Epigenomics and Roadmap Epigenomics Integrative Analysis (37). The Ensembl Regulation hub provides the first regulatory annotation on the new human hg38 browser. A public hub of long-read (454) RNA-seq for K562 and HeLa provided by Stanford complements ENCODE annotations on these intensively studied cell lines.

With the completion of UCSC's Data Coordination Center responsibilities for ENCODE, the ENCODE portal (encodeproject.org) was migrated to the new ENCODE DCC. The new portal hosts the latest data and access tools for ENCODE, as well as data from the first production phase of the project (2007–2012). UCSC continues to make available all ENCODE data collected and tools developed at UCSC during the pilot and first production phases (2003–2012) of ENCODE. The genome.ucsc.edu/ENCODE web pages have been updated to reflect UCSC's changed role.

#### Comparative genomics

The highlight of comparative genomics work at UCSC this year was the completion of two massive multi-species sequence alignment and conservation projects. The largest comparative track at UCSC to date is the 100-species Multiz Alignment and Conservation track on human assembly hg19. The pairwise alignment step alone consumed roughly 135 000 CPU hours and 4 months of elapsed time. The end product was the release of more than 74 GB of compressed file data. The same methods were used to build a 60-species Multiz Alignment and Conservation track for the mouse (mm10).

New tracks based on UCSC multiple alignments this year include phastBias-predicted regions of GC-biased gene conversion (38) and the Ultraconserved Elements public hub showing regions of the genome longer than 200 base pairs with 100% sequence identity in human, mouse and rat genomes, 99% conserved in dog and 95% in chicken (39).

The assembly hub browser feature was used to construct a comparative genomics public hub for bacterial genomes, featuring 57 *Escherichia coli* strains and nine other species (described in more detail in the Comparative Genomics section, below).

#### Other

##### Dog genome

The Broad Improved Canine Annotation (40) added to our public hub menu this year provides 40 data sets of annotation in the dog genome, including coding and non-coding genes, transcription by RNA-seq in multiple tissues and several SNP tracks.

##### Zebrafish genome

The Zebrafish Genomics public data hub contains data sets describing the NHGRI-1 zebrafish inbred line (41), which was developed for high-throughput genetics and genomics.

## NEW SOFTWARE FEATURES IN THE GENOME BROWSER

### General display enhancements

#### Stacked ‘wiggle’ display

**Figure 1.**
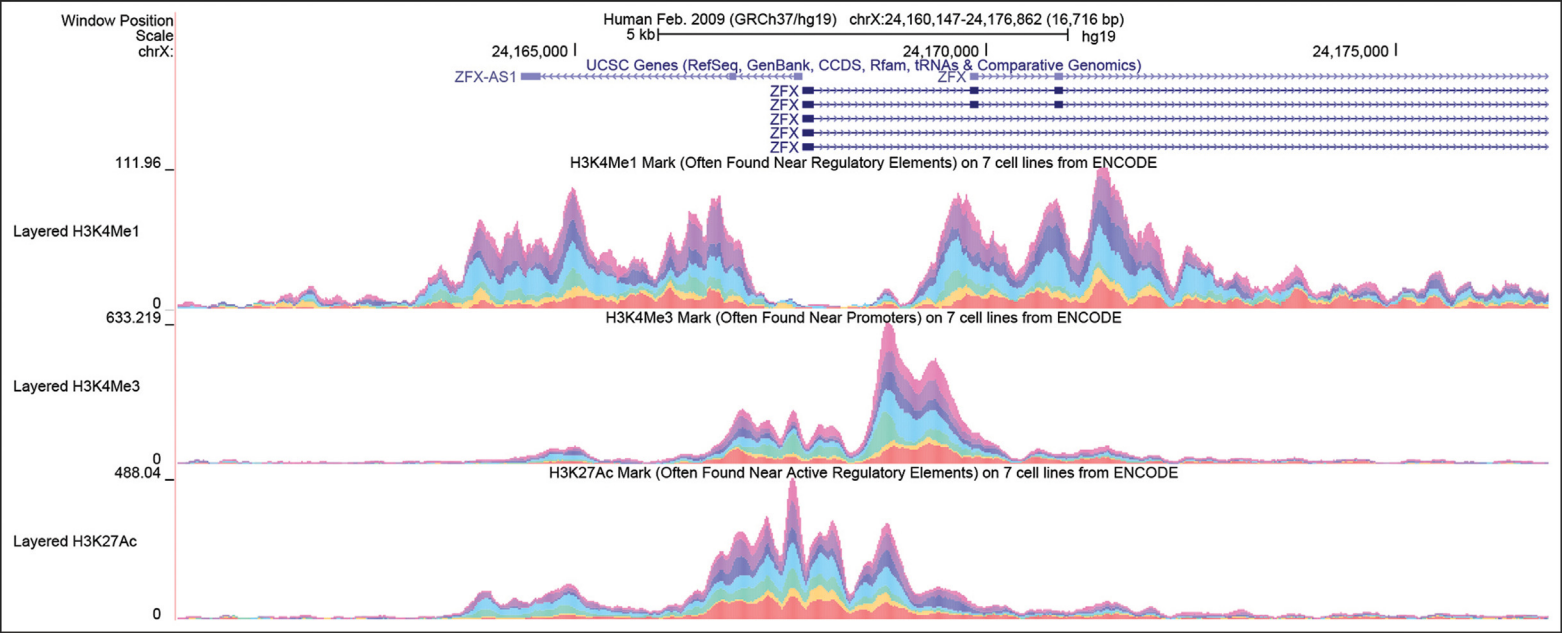
Genome Browser screenshot of the transcription start site of the ZFX gene illustrating the new stacked color graph display. The enrichment of histone marks H3K4me1, H3K4me3 and H3K27ac is shown in three browser tracks, where contribution to the sum of the signal by each of the six different cell types is indicated by color. Note that the K3K4me1 signal is low in all cell types across the promoter and at the beginning of the transcript, but high on either side of the promoter. The other two marks show the opposite tendency, being highest at the promoter and declining with distance from the promoter. In general the signals are strongest in the turquoise cell line (HUVEC).

A new track configuration option is now available for multi-wiggle (graph) tracks to complement the colored overlay display. Figure 1 shows examples of multiple annotations that are colored and presented vertically in an additive fashion in a single track.

#### Region highlighting

We have added a much-requested feature to highlight a genomic region in the browser image. The feature allows the user to click and drag across a region and select highlighting as an option (versus zooming in to the region).

### Specific annotation display enhancements

#### Transcription factor motifs

We have added a browser display feature to highlight transcription factor motifs in transcription factor clusters and to show motif information, including sequence logos, on the track item details page. This display is currently featured in the human hg19/GRCh37 ENCODE Regulation Transcription Factor track.

#### Hierarchical alignment format

We have added display support for Hierarchical Alignment (HAL) format, which uses a graph-based structure to efficiently store and index multiple genome alignments and ancestral reconstructions (42). To visualize HAL alignments in the browser, we developed a new ‘snake’ track display type that provides a way to view sets of pairwise gapless alignments that may overlap on both the chosen genome (reference) and the query genome, and shows all types of genomic variation, including substitutions, indels, rearrangements and duplications (43). Snake tracks are analogous to a linearization of the popular circular genome plots (44), but implement intelligent alignment scaling, making them useful at all levels of detail, from individual bases to whole chromosomes. Stacking multiple snake tracks allows a flexible view of the multiple genome alignment, elucidating structural variants between genomes. The snake track type is showcased in the *E. coli* Comparative Genomes hub described below. At present snake tracks for HAL visualization are available only for assembly hubs (see below), but we will be extending this feature for use in custom tracks.

#### RepeatMasker-nested elements

**Figure 2.**
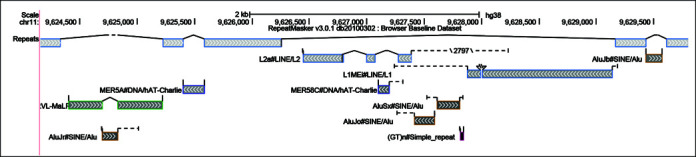
Genome Browser image of interspersed repeat (IR) annotation on a short region of the human assembly hg38 using the new visualization track. This region contains several layers of nested IRs, created when transposable elements insert into and break up older IR sequences. The solid lines join aligned fragments of an IR and can often reveal interesting features of a locus such as target site duplications. The dashed lines represent the unaligned portions of the transposable element sequence and allow one to visually decipher the relative consensus position of the fragments. When it is not practical to draw the unaligned sequence to scale, the length is displayed between two hash marks. Each annotation box encodes the class of the repeat using the colored edge, the divergence of the repeat with the grayscale interior fill color (lighter levels of gray indicate higher divergence) and the orientation of the repeat using arrowheads.

A new display of repetitive elements aims to visualize the complex relationships between interspersed repeats (copies of Transposable Elements) scattered throughout mammalian genomes. The previous visualization lacked the ability to view both singleton fragments and joined fragments in the same track, making it difficult to decipher the order of insertion for nested repeats. In the new display (Figure 2), when viewing a sufficiently small region, elements fragmented by nested insertions are connected by solid lines relating aligned fragments and model duplications or with dashed lines representing unaligned portions of the transposable element model. The new glyphs distinguish full-length insertions from partial copies, show the position of a fragment relative to the model, denote large-scale deletions and identify putative target site duplications. Labels now appear next to each element with identifier and classification details, and standard arrow notation is provided to indicate element orientation. Backing the new display are additional tables extending the annotation with model alignment details. Full RepeatMasker annotation and alignment information are presented on the track details page, and the table data is available for download.

### Locating data of interest

We have expanded the search features on the Genome Browser website to facilitate locating data of interest. The track hub selection interface has been redesigned for usability and now includes a search box that can be used to list public hubs with descriptive pages that include the search term. Item search is now supported on browser tracks in bigBed format.

### Data Integration

During the past year we expanded the feature set of the VAI to support assembly hubs and added two new input options: the user can paste a list of dbSNP rs identifiers or can choose an automatically generated list of artificial example variants to enable experimentation with different options before plugging in real variants. To detect possible regulatory consequences of variants, we have added two ENCODE Regulation summary tracks to the list of optional data sources for hg19: DNaseI Hypersensitivity Clusters in 125 Cell types and Transcription Factor ChIP-seq for 161 transcription factors.

The Table Browser output options were expanded this year to include data export to GenomeSpace.

### Comparative assembly hubs

A comparative assembly hub is a collection of assembly hubs interrelated by a multiple sequence alignment that models all forms of structural change, including rearrangements and duplications. The underlying alignment can also be used to lift annotations between browsers in the hub, so that bigBed or bigWig annotations (45) on one browser can be projected onto another automatically. We have developed a pipeline to generate a comparative assembly hub featuring snake tracks from an HAL multiple alignment file (43). The HAL package provides a tool for building HAL files from an existing MAF format alignment, or alternatively the Cactus multiple alignment software package (46) can be used to generate an HAL alignment. We have applied the pipeline to an alignment of 67 strains of *E. coli*; the resulting *E. coli* Comparative Assembly Hub is available on the Genome Browser's public hubs page. Comparative assembly hubs are loaded into the Genome Browser in the same manner as other assembly hubs, by pasting the URL of the configuration file into the track hubs page.

### Software distribution

#### ‘Genome browser in a box’ (GBiB)

To make local Genome Browser installation simpler and to assist in protecting the confidentiality of sequencing data from human subjects, we have developed GBiB, a virtual-machine image of the Genome Browser (47). Based on Oracle VirtualBox open source virtualization software, GBiB can be run on all major operating systems. The platform features a small memory footprint and easy installation on a laptop. The GBiB installation includes the UCSC Genome Browser software, all required utilities and a basic set of human genome annotation data. Additional annotation data can be loaded on demand from UCSC via the Internet or can be downloaded or viewed from the user's local disk for faster access and data privacy.

The Genome Browser command-line utilities are now packaged and updated with each build (normally every 3 weeks), and are now available for our Windows users via the GBiB platform.

To improve security and privacy on our site we have switched to cryptographically secure web identifiers for user and session and have incorporated software safeguards to protect access by database query (SQL injection).

## FUTURE PLANS

### Expanding the hg38 human genome browser

Efforts to increase the number of annotation tracks and re-alignment of existing functional data sets from GRCh37/hg19 to GRCh38/hg38 are underway, with an emphasis on high-standard quality assessment before public release. Important new tracks will include comparative alignments from 100 species and regulatory data sets such as DNA hypersensitivity to aid ongoing functional studies.

### Gene sets, expression and pathways

We plan to update the UCSC Genes track for human (hg38) and mouse (mm10) and will continue to update the GENCODE genes tracks in both species. The GENCODE track on human assembly hg38 will be expanded to include extensive gene details similar to the UCSC Genes track. We also plan to add an NCBI Genes track to host all RefSeq alignments from NCBI (and distinguish from the UCSC RefSeq alignments track) and develop documentation to assist our users in the selection of the appropriate gene set for their use.

The NIH Gene Tissue Expression (GTEx) project is creating a comprehensive atlas of gene expression and regulation across multiple human tissues. With an end goal of 900 donors and 20 000 tissue samples, the project has generated data from 4502 samples (215 donors) representing 55 tissues as of September 2014. UCSC plans to incorporate these data into the browser in a variety of ways, e.g. new tracks displaying tissue expression levels by gene and allele-specific differential expression by SNP. We will also use these data to enhance existing tracks and tools, e.g. the genes detail pages and the Gene Sorter. This will be the first major upgrade of comprehensive human gene expression data in the Genome Browser since we incorporated the microarray-based GNF Atlas2 in 2009.

In recent years the volume and quality of peptide identifications from tandem mass spectrometry have increased greatly. Proteomics resources such as PeptideAtlas (48,49), GPM (50) and ProteomeXchange (51) provide centralized data and metadata collection, uniform spectrum mapping and quality filtering for experimental results from diverse studies. To complement our gene sets and expression data, we are investigating how best to incorporate these data in our human genome browsers.

### Software

We plan to provide the GBiB in a format installable on major cloud service providers. Planned usability changes include adding exon numbering to mouseover on gene tracks and modernizing the website look and feel. We are developing a graphical network browser that will integrate text-mined interactions with major pathway and protein–protein interaction databases.

The Global Alliance for Genomics and Health (GA4GH) is an effort to create open standards for genomics (52). As part of this effort, web application programming interfaces (APIs) for sequencing reads and genomic variants are being established, with an initial v0.5 API specification finalized in August 2014. These APIs, supported by a number of data providers including the NCBI and EBI, will potentially provide another source of genome data that can be viewed and explored using the Genome Browser platform.

## CREATING A GENOME BROWSER

The construction of a new Genome Browser and database begins with the download of nuclear and mitochondrial genome sequences (fasta format) and assembly files (usually AGP format) from NCBI and GenBank. After verifying the sequence and assembly files for consistency, chromosome sequences are created and compressed into UCSC ‘2bit’ format.

A UCSC assembly ID is assigned using the first three characters of the genus and species, plus a number (for earlier assemblies, just the first character of genus and species) (53). Additional configuration items are the sequencing center identifier, NCBI genome, assembly and project identifiers, GenBank assembly accession, scientific name, common name and GenBank taxonomy identifier.

The first database tables and display tracks created for a new genome are those in the ‘Mapping and Sequencing’ browser annotation group. These include chromInfo (chromosome names and sizes), chromosome bands (displayed as an ideogram), assembly and gap detail, sequence quality scores and GC percent computed in a sliding 5-base-pair window.

Next, the chromosome sequences are analyzed for repetitive elements using RepeatMasker (A. F. A. Smit *et al.*, RepeatMasker at http://repeatmasker.org) (54) WindowMasker (55) and Tandem Repeats Finder (56). Results from these tools are used to mask the sequences so downstream analyses such as whole genome alignments can bypass or otherwise adapt to this prevalent component of vertebrate DNA. Details of the repeat analysis are saved in the database and used as the basis of visualization via tracks in the Variation and Repeats browser annotation group (RepeatMasker, Interrupted Rpts and Simple Repeats).

Sequence-based analysis tools are used to create basic gene and regulatory annotations. Cytosine-phosphate-Guanine dinucleotide (CPG) islands are annotated using the Miklem and Hillier method (57) to create a first transcriptional regulation track. The GENSCAN gene finder (58) is applied to create a basic gene annotation. Functional annotation from experimental evidence begins with alignment of RefSeq genes, mRNAs and ESTs from GenBank. For biomedically important and other well-studied genomes, gene models, gene expression and gene regulation annotation groups are filled in with experimental and curated data sets.

Finally, new genome assembly sequence is aligned to other species using the UCSC/Penn State University comparative genomics pipeline. A low-coverage assembly may be used in a multiple alignment for a closely related organism, and most assemblies have a track prepared for human. For important genomes, a multiple sequence alignment and determination of evolutionary conservation across the genome are prepared using lastz (R.S. Harris, unpublished thesis), chain/net (18), multiz (59) and conservation tools from the PHAST package (60).

The final steps before public release are the creation of a BLAT server for sequence searches, and completion of descriptive web pages for the assembly and annotation tracks. The finished browser is then reviewed in depth by the UCSC quality team, using automated and manual testing. New genome browser releases are announced on the Genome Browser announcement mailing list, posted on Twitter and featured in the News section on the browser home page.

Detailed documentation about the UCSC Genome Browser build process and comparative genomics pipeline can be found on the UCSC Browser GenomeWiki (genomewiki.ucsc.edu).

## CONTACTING US

Questions can be directed to our public mailing list (genome@soe.ucsc.edu), but we request you first search the list archives to see if your question has already been answered. For notification about new features and data at our site, join our announcement list (genome-announce@soe.ucsc.edu) or follow @GenomeBrowser on Twitter. Complete contact information, links to the list archives on Google Groups, information about training seminars and access to our user suggestion box can be found on our Contacts page (http://genome.ucsc.edu/contacts.html).
